# Comfort and challenge in the classroom: exploring the role of safety in sex- and gender-sensitive medicine education

**DOI:** 10.1186/s12889-025-23444-2

**Published:** 2025-07-02

**Authors:** L. Modderkolk, L. Van den Hurk, S. Oertelt-Prigione

**Affiliations:** 1 Gender Unit, Department of Primary and Community Care, Radboudumc, Radboud University Medical Center, Geert Grooteplein 21, Nijmegen, 6500 HB Netherlands; 2 AG 10 Sex- and Gender-Sensitive Medicine, Medical Faculty OWL, University of Bielefeld, Morgenbreede 1, Bielefeld, 33519 Germany

**Keywords:** Sex, Gender, Medicine, Teaching, Transformative learning, Education, Multi method

## Abstract

**Background:**

There is growing focus on sex- and gender-sensitive medicine (SGSM) in the field of medical education, yet only few medical schools worldwide have successfully integrated SGSM content into their curricula. The topic is highly relevant, but it can be challenging due to its identity-related nature that can trigger questions about safe learning spaces. In this study we aimed at investigating the multilayered impact of safety on SGSM teaching.

**Methods:**

We employed a multimethod qualitative approach to investigate perceptions and experiences of safety in studying SGSM and the implications for curriculum design. In the setting of a week-long summer school on SGSM we performed participant observation, a creative assignment, a focus group, and semi-structured interviews with participants to achieve a multi-dimensional framing of the concept of safety.

**Results:**

The study highlights the interrelation of psychological, social, and cultural safety and a hierarchy in their exploration by the learners in the context of SGSM. If social safety is not guaranteed, learners do not proceed to explore psychological and eventually cultural safety. Learners often conflated safety with comfort and avoidance of conflict. Placing an explicit focus on safety can, thus, have a paradoxical effect, with learners avoiding learning about sensitive topics because of a heightened attention to safety.

**Conclusions:**

Conflation of safety with conflict avoidance can hinder learning in the field of SGSM as it might hinder deep engagement with the topic. Lecturers are responsible for the generation of safe learning spaces focused on social, psychological and cultural safety, yet need to encourage exploration and discussion within this framework. Hence, lecturers need appropriate training to support safety, in-depth engagement and critical reflection in class, in order to promote personal growth while acquiring professional skills in SGSM.

**Supplementary Information:**

The online version contains supplementary material available at 10.1186/s12889-025-23444-2.

## Background

Recognition of the impact of sex and gender on medical practice and research as well as a greater awareness of structural inequities within the healthcare system [1, 2] are leading to a growing demand for the incorporation of sex- and gender-sensitive medicine (SGSM) content into medical curricula. Especially medical students express a desire for training in SGSM, recognizing its relevance for their future professional role [3, 4]. SGSM focuses on policies, programs, or interventions in medicine that explicitly address biological sex differences as well as culturally defined gender roles, duties, rights, responsibilities, and accepted behaviors [5].

Some medical schools around the world have successfully incorporated SGSM content into their mandatory teaching programs [6, 7]. However, systematic incorporation into medical curricula is still rare. Since sex and gender are fundamental components of human identity, it is necessary for future physicians to learn about the complex interplay between biological mechanisms, diagnosis and treatment [1] and the role of gender in communication and action [8].

Teaching about identity-related aspects and discrimination in medicine poses several challenges, as described in the recent literature about race and racism in medical curricula [9–11]. This can be attributed to a historical focus of medical curricula on biomedical rather than psychosocial aspects, as well as the discrepancy between knowledge and skills provided in medical education and real-world medical practice [12]. Teaching about race and gender is challenging because of their complexity and nuance, their social construction, their multilayered impact on health, and the difficulty to quantify critical learning outcomes with standard methodology [13].

A safe and inclusive learning environment is paramount for the learning process about sex and gender in medicine. Safety is an important, yet often overlooked, contextual factor in the learning process of sensitive topics like SGSM [14–16]. Emphasizing the importance of safety recognizes the personal and potentially sensitive nature of the topic, and can foster an environment that encourages open dialogue, self-reflection, and respectful exploration of diverse perspectives. This approach not only enhances the educational experience but also contributes to the overall well-being of learners, teachers and patients involved in the study of sex and gender in medicine. However, the term"safety"is commonly used in medical education without a precise definition to optimize its role and interpretation. This study aims to identify which types of safety are relevant to studying SGSM, their interactions and how safety can be enhanced while teaching SGSM.

## Methods

### Design

**Fig. 1:**
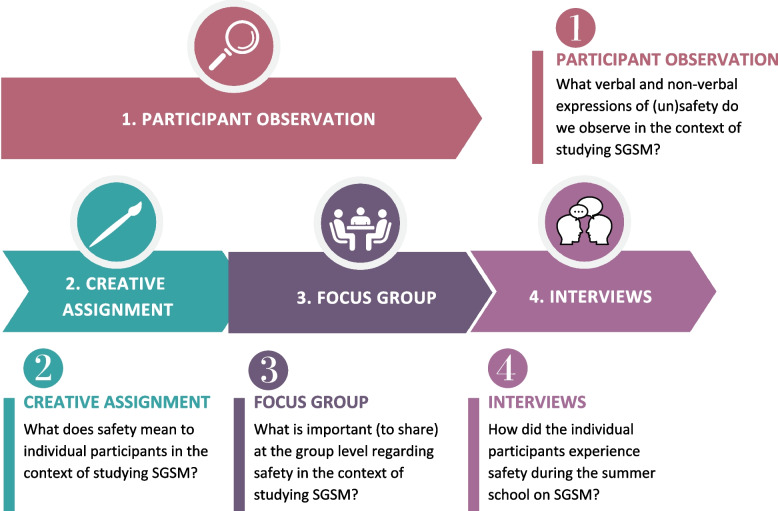
Flow diagram depicting the different data collection moments

A multimethod qualitative study design was used to explore how participants conceptualize and experience safety while studying SGSM, and the implications for curriculum development. During a week-long summer school about sex- and gender-sensitive healthcare we performed (a) participant observation, (b) a creative assignment, and (c) a focus group to explore the topic of safety. In the two months following the summer school, we also conducted (d) semi-structured interviews with the individual participants (Fig. 1).

### Participants

Participants included professionals and researchers in the field of health and advanced medical students who joined the week-long international summer school ‘Towards individualized care: sex and gender perspectives in health’, which was part of the summer school program of Radboud University in Nijmegen in 2022. Upon registration for the summer school, participants received an information letter about the research linked to the summer school and an informed consent form, which they signed on the first day of the course. Refusal to participate in the research project did not lead to exclusion from any activities during the summer school. The Medical Research Ethics Committee Oost-Nederland evaluated the research, concluding this study was not subject to the Medical Research Involving Human Subjects Act (2022–13830).

### Data collection

#### Participant observation (1)

During the five-day summer school program, two researchers (LvdH and LM) observed the participant´s actions and verbal expressions without interfering with the program. All participants were informed about the research and the presence of the researchers. The researchers independently made notes, which they then compared at the end of the day to ensure the completeness and to minimize personal bias. At the end of the summer school, the field notes were combined into one document.

#### Creative assignment (2) and focus group (3)

As part of the summer school program, we performed a 75-min focus group with all participants on the topic of safety in studying SGSM. Participants were informed before the start of the summer school about the focus group's aim and allowed to ask questions about it until the beginning of data collection. Participation in the research was voluntary and participants were informed that unwillingness to do so would not preclude them from attending, their input would simply not be included in the analysis. All participants gave informed consent to participation and recording. Before the focus group, the participants individually engaged in a creative assignment that allowed them to visually/artistically express their understanding of safety. They were offered drawing and writing material, as well as magazines and newspapers, which they used to produce e.g. drawings, collages, and poems. Thoughts and insights gained during this assignment were reported during the focus group, which was audio recorded. The focus group was led by the facilitator of the summer school (SOP) and one of the researchers (LM).

#### Qualitative interviews (4)

Individual interviews with all participants were conducted upon completion of the summer school over a two-month period. These interviews lasted around 30 min, were performed by one of the researchers (LM) using video conferencing, and were audio recorded. The interviews focused on participants'personal experience of safety in studying SGSM during the summer school (for the interview guide see Additional file 1).

### Data analysis

All qualitative data were transcribed verbatim and analyzed using directed content analysis [17]. The safety types identified in our theoretical framework led to a first outline of the codebook and were enriched with the input from the observation notes. This codebook was used as a starting point for analyzing the focus group data and the interviews. Throughout the coding process, the codebook was supplemented with new codes. All data were read, coded, and discussed line-by-line by two researchers (LM, LvdH) using Atlas.ti 9 software until consensus was achieved. Following this first step, categories and themes emerging from the data were defined and followingly discussed and adjusted in a peer review session with the three researchers (LM, LvdH, SOP). This led to the development of an affinity diagram which was again discussed in a peer review session with the same researchers (LM, LvdH, SOP) and adjusted based on their feedback. The Consolidated Criteria for Reporting Qualitative Studies (COREQ) checklist was used for data reporting [18].

#### Research team positionality

In our exploration of the importance of safety in SGSM education, our research team acknowledges the profound influence of our own positionality on the inquiry process. As women with predominantly white, cisgender identities, we recognize that our backgrounds may have inherently shaped our understanding of the material. We recognize that our social positions may influence how we perceive and interpret what constitutes a"safe"learning environment, as well as how safety is experienced by learners with different identities than our own. Despite this, we are committed to critically examining how our privileges and biases intersect with the complexities of sex and gender in healthcare. Our team's diversity, both in professional expertise and personal experiences, allows us to approach the research with a nuanced perspective. By acknowledging and interrogating the impact of our identities, we strive to enhance the inclusivity and integrity of our work, ultimately contributing to the advancement of equitable healthcare practices for all individuals, regardless of their sex or gender identity.

### Theoretical framework

In this study we focus on three types of safety: psychological, social and cultural safety. The different types and their theoretical frameworks are described below, as well as their operationalization in this study (Table 1).

**Table 1:** Operationalization of the three safety types

Safety type	Definition	Level of observation
Social safety	‘Experiencing social connection, social belonging, social inclusion, social recognition, and social protection within the group’	Experience of belonging
Psychological safety	‘Displaying learning behaviors like speaking up with ideas, questions, concerns, or mistakes as a result of the belief that one will not be punished or humiliated for it and that the group is safe for interpersonal risk-taking’	Learning behavior
Cultural safety	‘Open and ongoing self-reflection to understand one’s own assumptions, biases, and values that (unconsciously) contribute to social injustices in healthcare and the systems that maintain them’	Reflective behavior

#### Social safety

Social safety refers to reliable social connection, social belonging, social inclusion, social recognition, and social protection, and it serves as the foundation for human functioning, as well as for exploring new territories and topics [19]. Marginalized individuals, such as those who identify as sexually or gender-diverse, often live in a constant state of threat-vigilance due to the stigma surrounding their identity [20]. This experience is known as minority stress, and results from the chronic, cumulative stress associated with stigma due to objective events, such as discrimination and victimization, and psychological responses to these events, such as internalized shame [21]. In every social situation, the essential question related to social safety is: “Is it safe for me here?” The word “here” emphasizes that perceptions of safety depend on local conditions, and on the specific constellation of physical, auditory, visual, tactile, and symbolic cues of safety and threat that are perceivable in a particular setting [19]. The “here” can be anywhere, but from an educational perspective it is especially relevant in settings where marginalized identities are explicitly discussed in the teaching context or where their existence becomes relevant for access to learning spaces.

#### Psychological safety

Psychological safety refers to how people perceive the potential outcomes of taking interpersonal risks in a particular context, such as the workplace [22]. Although it has been mainly studied in the context of long-term work-related groups to understand the dynamics of learning behavior, collaboration, and team performance, these dynamics are relevant in any group setting where learning occurs [22]. Central to studies and analyses of psychological safety across contexts is the focus on the willingness to share ideas to contribute to a shared goal. Kahn proposed that psychological safety affects individuals’ willingness to “employ or express themselves physically, cognitively, and emotionally during role performances,” rather than disengage or “withdraw and defend their personal selves” [23]. Our study focused on analyzing psychological safety at the individual level, operationalized as ‘interpersonal risk-taking in a learning environment'.

#### Cultural safety

Cultural safety involves healthcare providers being aware of their biases, stereotypes, assumptions, attitudes, and systems that hinder health equity, and taking action to address these issues [24]. While cultural safety initially focused on racialized injustice in healthcare, it can also be applied to sex and gender inequalities present in the medical system and human interactions.

## Results

### Participants

In total 14 participants registered for the summer school. One had to cancel a few days before the start of the summer school due to personal circumstances and one participant returned home after the second day due to illness, which led to exclusion from the data collection. All 12 remaining participants (Table 2) gave written consent to participate in the research on the first day of the summer school, and verbal consent was recorded at the start of the individual interviews. Despite a diversity in professional backgrounds, all participants were actively involved in healthcare practice and/or research.

**Table 2:** Characteristics of participants

Characteristic	Number
**Gender identity (n)**	
Women	11
Men	0
Non-binary	1
**Age (n)**	
20–24 years	2
25–29 years	5
30–34 years	2
35–39 years	1
40–44 years	1
50–55 years	1
**Ethnicity (n)**	
German	5
Italian	2
Spanish	3
Portuguese	1
Filipino	1
**Profession (n)**	
Healthcare researcher	5
Researcher/physician	1
Researcher/psychologist	1
Researcher/lawyer	1
Medical student	2
Entrepreneur	1

### The relevance of safety in studying SGSM

According to the participants, safety is essential for learning about SGSM. While safety is vital in all learning contexts, participants emphasized the added relevance of safety in studying SGSM, given the perceived sensitivity of the topic. According to participants, the goal of studying SGSM within a group setting was ‘to increase mutual understanding due to the diversity of experiences and opinions’ in which safety was framed as ‘an open and non-judgmental environment where non-confrontational interactions can occur’.

*‘Safety is really important because we are dealing with people from different backgrounds and with different identities, so maybe not all of them are out or out with it in the open, so they have to have a safe space to communicate with people they trust, and feel comfortable talking to. I guess, yeah, safety is important, especially with sex and gender.’* (R10)

### Relevant safety types for SGSM

The data analysis focused on exploring the relevance of three types of safety for studying SGSM: social, psychological, and cultural safety.

### Social safety

#### The experience of social safety

Participants’ experiences of social safety varied and were influenced by distinct social identities and temporal considerations. Social identity dimensions such as age, gender identity, ethnicity, and religion appeared crucial in shaping participants’ perceptions of safety. Most participants looked for indicators of social safety by focusing on various cues, including the room’s arrangement, other participants’ facial expressions, introductory remarks by course leaders, and shared personal stories.

*‘This welcome exercise you chose, you made us open up because we got to share the story of our names. Of course, opening up and telling something personal brings people closer together because we can see a glimpse of personal stories.’* (R6)

The multifaceted social identities that affected safety levels were noticeable in the evolving social roles that participants took on throughout the course. These roles included personal, professional, advocate, learner, outsider, representative, senior, mediator, group member, and teacher. They subtly guided participants'attitudes and behaviors, often without explicit acknowledgment. However, participants consistently looked for interpersonal commonalities, affirming their social safety and cohesion within larger and smaller group dynamics.

#### Exploration of social identities

The perceived and identified gender identities of the participants did not always align, but this matter was largely undisclosed or undiscussed. While most expressed feeling socially safe during the course, two participants had a different experience that they attributed to their sexual or gender-diverse identity. One participant purposefully chose not to disclose herself as queer, while another expressed her non-binary identity yet felt others didn’t pick up on it. One of these participants reported that their learning abilities during the course were affected by this experience.

*‘The difficulty in the week was when people kept saying: ‘we are all women in this room and all of us are women’. The fact they say we are women and everything inside was like: no no no no. I just completely zoned out when that happened, and I wasn’t following anymore, and that made me really angry at times, because I wanted to be there and be present, but I couldn’t in those moments, but I also couldn’t say anything. It’s a time when you are the only person to say: ‘hold on, it’s not true’. ’* (R3)

*‘I know that I was trying to be extra careful not to harm anyone, so I was avoiding a confrontation on purpose regarding that because we never talked openly at the beginning about different views that people may have, and I could understand that we didn’t have sex diversity, but we had gender diversity, but we never talked openly about that.’* (R5)

Discrimination and stereotyping emerged as relevant factors in the context of diversity, particularly concerning gender identity. However, these were only briefly touched upon from a theoretical standpoint but not observed or addressed in the mutual interaction. A few participants expressed concerns about the perceived safety of groups with greater gender diversity, especially for women in the presence of men. Instances of unspoken gender assumptions within the group underscored the complexities associated with navigating diverse identities.

*‘Because I was wondering if the results or experience would change if in the class there is a combination of different genders, male and female, in terms of sex, not only females or non-binary persons.’*

*I: ‘What makes you think that?’*

*R: ‘Basically because in my case, I think usually I’m feeling not as comfortable if they are all male, not with all of them for sure, but if they are in this superior position, you cannot express in the same way in the environment that is different.’* (R2)

Participants reported varying preferences regarding the balance between exploring each other's perspectives and engaging in constructive debates. Several participants mentioned there was too much emphasis on avoiding hurt feelings, which limited productive discussions. Even though learning about SGSM occurred, richer learning would have been possible, according to some. Others felt it was unnecessary to explore personal identities and related stories.

*Interviewer: ‘Do you feel that for you or in the group, there have been moments where the focus on being careful went sometimes at the expense of daring to openly discuss topics that might feel a bit more unsafe or uncomfortable? I see you nodding.’*

*Participant: ‘Yes, I think that part was missing. I think we need that additional layer that I think it would be uncomfortable at the beginning, but then we would be in the real world, because that was a very safe environment and I really missed that, so that would be something that I would like to have been exposed to, because I think it would have been challenging. I think that would have felt uncomfortable, but it would be a gift, because then I would be best prepared for.’* (R5)

#### Suggestions for social safety

Teachers play a pivotal role in shaping participants' experiences. Some participants highlighted the importance of a proper introduction, connection-building, and ground rule establishment deemed essential for fostering social safety. Suggestions were made to enhance social safety by explicitly addressing specific topics, like pronouns, not just at the outset but revisiting them opportunistically during the course. One participant also emphasized the importance of reducing uncertainty through participants sharing diverse backgrounds at the beginning.

### Psychological safety

#### Perceived preconditions for psychological safety

In the context of psychological safety, one of the most essential factors mentioned by participants was feeling emotionally comfortable. Various factors, such as individual experiences, interpersonal interactions, specific topics discussed, and exercises conducted, influenced this comfort. It significantly impacted participants'willingness to engage, learn from mistakes, and express themselves freely—including voicing their opinions and perspectives. While emotional expression was considered vital, it was also perceived as potentially risky.

Some interactions led to an increased mutual understanding, facilitated by what participants described as an ‘open and non-judgmental atmosphere’. These facets of the learning environment were deemed indispensable for fostering a sense of safety among participants. During the week, as participants got to know each other, the sense of safety increased, resulting in more self-expression and interaction within the group.

*‘I think there is an aspect of feeling safe in itself, of what you do think, and to be secure to express it and think that the others will accept that opinion or at least leave the space to express it or to allow different opinions. To have like certain space, to be able to express thoughts, feeling without being judged or that make you feel little and that you cannot express them.’* (Focus group)

*‘The peers were really important to see that kind of diversity and also hearing them expressing themselves and also myself was safe to express my points of view, which were different or even in that antagonistic sometimes, but that's part of the diversity and inclusion.’* (R11)

#### Learning behaviour

Although the atmosphere was experienced as open and non-judgmental, most participants avoided discussing potentially controversial topics or expressing strongly diverse viewpoints. As a result, discussions were kept non-confrontational and were marked by a lack of active exploration of differing perspectives. Ironically, the perception of safety as synonymous with feeling comfortable led participants to feel responsible for ensuring the comfort of others. Some participants acknowledged that they were hesitant to discuss contentious topics due to the fear of unintentionally offending others and, therefore, refrained from asking questions or sharing their perspectives.

*‘I must say in the context of sex and gender and all these topics that are, of course, sometimes sensitive, I think I'm sometimes a bit afraid, or I don't know if I can say afraid, but a bit cautious that I said something wrong or do something wrong.’* (R9)

*‘I felt from my own and I could see that some people would not express everything because we were all a bit afraid of being too confrontational at the end. [...] I think there is this misconception that confrontation is bad, and I don’t think it is bad if it is done properly. [...] It’s true, I hold back a lot of times.’* (R5)

Reluctance in engagement impeded discussions on sensitive subjects integral to learning about SGSM. Subsequently, people described constantly balancing their need for belonging (fitting in) and expression (standing out). Learning behaviors were therefore displayed less than participants would have wanted, leading to possible suboptimal learning outcomes.

*‘It can be a bit of a difficult topic [sex and gender, red.] and I also waited for this moment to have the clashes, but I don’t know if it’s because we all had the same opinion or if you are just always too polite.’* (R6)

#### Suggestions for psychological safety

Some participants suggested smaller group formats to enhance discourse and learning about sensitive topics. The proposal entailed delving into nuanced subjects within more intimate settings, subsequently sharing the insights gathered in the broader group context. These participants stated that small group learning promotes a more robust exchange of perspectives and facilitates a deeper exploration of sensitive themes in the SGSM learning experience.

*‘Sometimes it would have also been nice to have focus groups, smaller groups where you go into more sensitive topics, where people actually have a perspective, even a topic like, as I said domestic violence or some things that are more difficult to address, sexuality, child abuse by the way which is I think a huge issue as well. Other topics could have been treated in smaller groups. We didn’t have some more discussion, or we didn’t go into these topics, because it was always in the big group.’* (R7)

### Cultural safety

#### Perceived relevance of cultural safety

A few participants highlighted the significance of addressing diverse cultural perspectives and assumptions. These considerations spanned sex and gender, religion, and socio-cultural contexts reflective of the participants'varied nationalities. The desire for critical reflection on intersectional concepts stemmed from general perspectives or personal experiences.

*‘Which is the topic of religion and this spirituality part. I mentioned it. I sometimes feel in this context, where people are usually feminists, they are more secular. This is being a bit shunned because, of course, there are a lot of issues in terms of the rights of women and people of other genders. I know this, but it’s still a perspective that many people have.’* (R7)

Divergent perspectives on topics such as positive discrimination and the intersection of feminism with religious identity underscored the necessity of engaging with nuanced cultural perspectives. The instructors did not initiate such a discussion, and when a participant brought it up, it was not responded to sufficiently, as became evident from the interviews and participant observation. The lack of discourse on these intersectional themes left some participants dissatisfied, fueling a desire for more profound reflections on the cultural dimensions inherent in sex, gender, and intersectionality.

*‘As I mentioned, my family lives in (X) and people don’t even have a way to measure their pulse sometimes if you go to a hospital. I don’t want to dig into it, but the truth is, of course, a lot of these stories connect to me. It’s multiplied in many ways because if a person says, for example, my father was diagnosed with Parkinson and it’s really horrible for me, I understand that. Still, at the same time people in my home in Cuba right now there is a person with Alzheimer in my neighborhood. They took her into our home to take care of this person there and they don’t have any medication. It’s like a complete misery and you don’t want to even start on these topics, because it’s so emotional. [...] I felt people were not picking up the topic, but maybe it was just not their topic or maybe it didn’t relate to their personal experience or they just felt it was out of the scope of sex and gender things.’* (R7)

#### Suggestion to enhance cultural safety

The absence of meta-discussions on how intersectional concepts are interpreted in different contexts elicited the desire for more inclusive conversations in some participants. One participant suggested perspective-changing exercises through the integration of role-play in teaching methods.

*‘I had a course at university about sexuality. There, we did role play, and I was, for example, a very conservative Muslim, and I had to think in this role and express myself in this role. This was nice because, of course, it was not my opinion but then I tried really to think about, yeah, how would I feel if my son was homosexual and all of my family wouldn't understand this, and it's probably against my religious values. I think changing perspectives sometimes helps us to understand why people are behaving the way they are.’* (R9)

### Dynamics between the safety types

The data analysis revealed a dynamic relationship between various types of safety, particularly in conversations about sex and gender. Participants were greatly aware of the topic's potentially personal nature, leading to its perceived sensitivity and a need for careful interactions. Since most participants viewed safety as synonymous with a comfortable and secure environment, this often restrained the opportunity for open discussion and exploration of the issues, which paradoxically compromised safety.

**Fig. 2:**
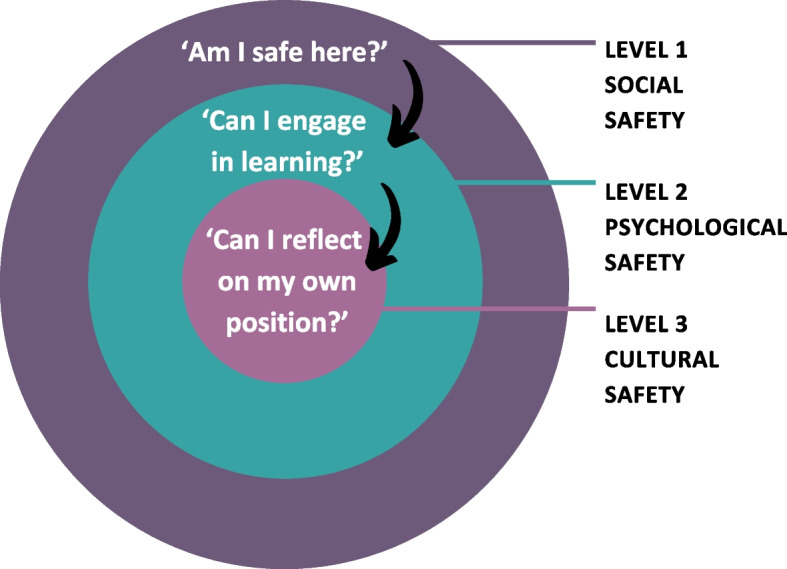
The three safety types and associated questions

Participants, often subconsciously, seemed to undergo a sequence of three interrelated inquiries during a SGSM learning experience that influences their safety perception and subsequent learning outcomes. This is visually represented in Fig. 2. The questions appear to be building upon each other in an almost hierarchical fashion. If a lower-level enquiry is not satisfied, the participant will not move on to the next level and the associated engagement and learning.

The first domain addressed is social safety, with participants asking themselves,"Am I safe here?". This question assesses their subjectively perceived social safety within the group, and whether their social identity is accepted. This is not a one-time question but a recurring one, dependent on changing situational contexts and topics. Only after receiving an affirmative response to the first question do participants proceed to the second inquiry:"Can I engage in learning?". Learning requires interpersonal risk-taking. Participants engage in learning activities exclusively when they feel psychologically safe to confront potential failure or diverse opinions—a condition that, like social safety, is dependent on the situational context they find themselves in. Lastly, a subset of participants poses the question,"Can I reflect on my position?"Having established a sense of safety that facilitates active learning, some individuals challenge both themselves and the group, encouraging a step back to reflect on their positionality and associated biases and assumptions. These reflections often span multiple, intersecting social identities, whose impact on the experience of safety can vary depending on the topic being discussed and which identities are made salient, either by the individual or through the dynamics of the group. As the data analysis showed, the interplay among the three safety categories is continuous and mutually reliant.

## Discussion

The study's findings shed light on the complex dynamics of safety in studying SGSM within a group context. The emphasis on psychological, social, and cultural safety reveals the interconnected challenges that participants face in engaging with this multilayered topic. While the study analyzes the impact of different types of safety, it also uncovers paradoxes in the participants'behaviors. Despite the creation of an open and non-judgmental atmosphere that most participants experienced as positive and helpful for learning, participants avoided potentially sensitive or controversial topics in the attempt to maintain a “safe” environment, which eventually resulted in non-confrontational discussions. This reluctance to engage in uncomfortable discussions hampered the depth of learning about SGSM. Hence, our study raises a number of questions that need to be addressed at different structural levels to optimize the SGSM learning experience. These questions and associated suggestions are based on our findings and relevant literature and educational practice and are presented below (Table 3).

**Table 3:** Structural questions about safety at different levels of the SGSM learning process

	Student level		Teacher level		Institutional level	
	Questions	Suggestions	Questions	Suggestions	Questions	Suggestions
**Social safety**	• Am I safe here? • Who is in the room? • Can I/should I share my identity/identities?	• Co-create group agreements; clarify that safety is not comfort but respectful learning • Use varied introductions to build relational awareness and broaden the understandings of identity [25, 26] • Normalize voluntary sharing; offer anonymous/creative formats	• How do you set the environment to guarantee social safety? • How do you navigate different/conflicting safety needs? • How do you feel safe yourself as a teacher?	• Co-create group norms; model respectful, inclusive behavior • Acknowledge tensions; use structured dialogue to hold multiple perspectives [26] • Seek peer support; set your own boundaries transparently [27]	• Which institution-wide rules and practices are put in place to facilitate social safety for students and teachers?	• Adopt inclusive language and policies (e.g., name/pronoun use, inclusive forms); enforce clear codes of conduct [19] • Establish diversity across all staff levels [28]
**Psychological safety**	• Can I engage in learning? • Can I ask more about […]? • Is it ok not to engage in/share […]?	• Use low-threshold activities (e.g., think–pair–share) • Provide anonymous question options; encourage peer dialogue • Affirm different engagement forms; offer private reflection options (e.g. journalling)	• How do you engage students in learning about identity-related topics while protecting their individuality? • How do you balance the safe and the brave space?	• Use consent-based participation; offer varied ways to engage (oral, written, visual) • Clarify that discomfort can be productive; frame conflict as part of learning by introducing ‘Brave Spaces’ [26]	• Which resources do you provide teachers and students with to support psychologically safe learning and working? • How do you address potential conflicts between students and teachers regarding safety?	• Provide staff/student training in identity-conscious teaching • Offer (independent) mediation services and transparent reporting channels for handling safety concerns
**Cultural safety**	• Can I reflect on my position? • Can I invite others, including the teacher, to reflect on their position?	• Frame reflection as a continuous and professional practice • Use tools like identity wheels or guided (privilege) reflection prompt • Teach constructive feedback (e.g., sentence starters, feedback protocols); model mutual reflection	• How do you facilitate critical reflection and consciousness? • How do you constructively navigate diversity experiences and perspectives? • How do you position yourself in the context of a diverse cultural setting?	• Use guided reflection prompts; connect personal experiences to systemic structures • Encourage dialogue across difference; validate lived experiences without essentializing [19, 29] • Be explicit about your own positionality and biases; invite reciprocal reflection [30]	• How can the institution create a diverse and inclusive learning environment? • How does the institution reflect on its own positionality and unconscious bias? • What rules and practices are put in place to invite students and teachers to offer feedback on the organizational positionality and unconscious bias?	• Ensure diversity in faculty/staff hiring; embed SGSM and equity topics in all curricula [19, 28] • Conduct regular curriculum and policy reviews with equity-informed external advisors • Create feedback loops (e.g., advisory councils, anonymous surveys) involving students and staff [31]

### The need for safety in learning SGSM at the student level

Our research reveals that a subjectively perceived insufficient degree of social safety [19] had a negative impact on the learning behaviors and outcomes of some participants in this setting. Medical education often overlooks the affective component of learning, which belongs to the domain of social safety, despite its significant impact on academic performance [32]. Maslow's hierarchy of needs, a classic theoretical framework to define necessities for human flourishing—physiological needs, safety needs, love and belongingness needs, esteem needs, and self-actualization needs—addresses these affective prerequisites. According to Maslow, needs can be divided into"deficiency needs"that need to be met before individuals can devote their attention to"growth needs” [33, 34].

Maslow´s model bears many analogies to our findings: if the “deficiency need” of safety is not fulfilled, learners cannot proceed to meaningful further engagement towards self-actualization. This is especially disadvantageous for participants in medical education who identify as sexual and gender diverse [35], however, our analysis showed that all participants were affected by social safety in the learning space. Driven by different motivations, most participants were monitoring the group for safety signals, e.g. whether it was appropriate to engage with a certain topic or present an opinion, constantly engaging in safety-maintenance strategies [19]. While these careful considerations might seem thoughtful and conflict-minimizing and align with their understanding of safety, they do not necessarily create an experience of safety for all participants. The derived insecurity for some participants emphasizes Diamond and Alley´s statement that ‘the only way to interrupt this preparatory vigilance is through explicit indicators of social inclusion and protection’ [19] which starts with a shared and expanded understanding of safety and associated learning behaviors. Teachers or facilitators play an essential role in this regard.

### Addressing safety needs in SGSM education by teachers

Explicitly investigating safety requirements among the participants highlighted the complexity of addressing divergent needs in a diverse group. Several participants preferred a more prominent role of the teachers in navigating challenges around safety and learning about SGSM. However, the interpretation of this varied. Some participants mentioned that it would have been helpful to make certain aspects, like pronouns, explicit at the start of the course and not just in the introductory exercise. Others, however, prefer limited personal reflection exercises. Exposing participants to mandatory sharing of their identities and opinions might contradict the goal of creating a safe learning environment, as it could create tokenism [36] or force a ‘continuous coming-out’ process for those having underrepresented or marginalized identities [35]. The question then becomes: what is the ultimate goal of creating a safe SGSM learning environment?

Some argue that safety is the counterpart of real learning, suggesting that fostering ‘brave spaces’—where discomfort and challenge are embraced—is more conducive to meaningful educational growth than striving for entirely ‘safe spaces’ [25]. These insights are inspired by, for example, Leonardo and Porter who suggest that"liberating violence"should be used in racial pedagogy and race dialogue, which is an identity-related theme similar to SGSM [26]. Their work, grounded in the thinking of Frantz Fanon [37] highlights the unique positioning and communication of people in majority or minority groups. Those in the majority group tend to intellectualize racism and are often unaware of their investment in the status quo. At the same time, those in the minority group have lived experiences being in a minority position. These different starting points can result in communication between groups becoming silent or agitated, with emotional responses from the minority group often labeled as distancing moves. However, Leonardo and Porter reframe this as emotional expressions being acts of engagement that, when guided carefully, can foster mutual understanding and learning. We have modified their suggested three steps for teachers to increase opportunities for racial education to apply them to teaching SGSM. These modifications can be implemented in either a week-long course, such as a summer school, or in a modified version in a regular seminar setting.

First, acknowledge that we are *all* gendered beings who are subject to gendered roles and norms. This removes the focus from the sexual and gender-diverse (SGD) students. Explicitly recognizing different gender dimensions can help broaden students'understanding of the concept and make them aware of their own biases, assumptions, and (situational) privilege [38]. Secondly, start the course with a meta-dialogue and reframe the classroom as a place of risk instead of safety. Paradoxically, emphasizing safety often is only safe for those already holding privilege. Therefore, it is recommended that students practice critical education and be encouraged to be curious and engage in discussions without shying away from friction. Critical reflection on self-understanding should be encouraged, as well as the option to change one's opinion. Sincere engagement, even when tense, honors the complexity of being human instead of confining people to one defining identity. Tools such as the academic wheel of privilege can help facilitate conversations that bridge intellectual perspectives and lived experiences. In this way a place of risk promotes growth, not hostility. This aligns with the desire expressed by a few respondents for cultural safety or reflection on one’s positionality. Lastly, identity-related teaching cannot exist in isolation and is always influenced by the people and systems around it. Therefore, the SGSM teaching institution should be mindful of the standards it holds itself accountable to. It should strive for the same level of rigor and excellence with regard to gender equity as it does for its other academic pursuits.

### Implications for medical education at the institutional level

Although our study does not focus on the institutional perspective, the previous section highlights its importance. Research indicates that students'gender identity significantly affects their experience in medical education. For instance, faculty members tend to unconsciously overlook the needs of LGBTQ + students and patients [39, 40] and SGD students determine their specialty choice partly based on their perception of the level of inclusiveness in the specialism [35]. Therefore, it is crucial to recognize the institutional perspective.

Implications can be drawn both at the program and system level to promote inclusivity and equity in medical education. At the program level, a SGSM perspective should be integrated throughout medical education, highlighting the impact of sex and gender components on illness and health [6, 7]. This should be accompanied by a critical pedagogy tailored to the different teaching formats. At the system level, teachers should be trained to facilitate the program-level ambitions. Diversifying the staff to reflect the population is a good starting point, but creating a fair and equitable learning environment is equally important [28]. Supporting students while building a more equitable system could be done through the mentorship of SGD students by SGD staff [27]. Institutions should take explicit steps to promote inclusivity, such as using SGD-affirming statements and symbols, asking people’s pronouns and using them correctly, and portraying SGD individuals in communication and educational content [19]. This will help create a more inclusive and safe learning environment.

Integrated approaches that operate across all three levels highlight what is possible and the impact that can be achieved, as well as the persistent challenges involved in putting these efforts into practice. These include managing differing expectations among stakeholders, the limitations of applying mechanistic problem-solving models to complex social issues, difficulties in assessing inclusion initiatives with traditional methods, navigating tensions between competing values such as social justice, tolerance, and civil discourse, and setting clear boundaries for respectful dialogue while fostering diverse perspectives [31]. To encourage more gender-diverse participation in SGSM programs, future efforts could focus on integrating SGSM content into standard medical curricula—promoting early and broad engagement—and developing specialty-specific training that highlights relevance to various medical fields. Together, these strategies may attract a wider range of participants and foster more inclusive learning environments.

### Future research

Future research could build on our findings and employ a combination of qualitative and quantitative methods to understand exactly how a supportive learning environment interrelates with the content, in the context of SGSM as well as other health topics shaped by social factors. Exploring these combined approaches in a variety of institutional settings and academic disciplines—especially those that engage with themes of identity, equity, and inclusion—would help clarify how local policies, cultural dynamics, and student backgrounds influence what works and what needs to be adapted. It would also be useful to test specific teaching strategies, like role-play exercises, regular reflection activities, or inclusive language practices, to see how they support both safety and learning. For longer courses or clinical internships, future studies should look at how these strategies can be adjusted over time—for example, how to maintain small group discussions, support facilitators, or integrate these practices into the overall curriculum. Comparing experiences in voluntary versus required courses could also offer insight into how student motivation affects the impact of these approaches.

### Strengths and limitations

The study has several strengths, one of which is the diverse methods used to collect and connect data during analysis. This resulted in a comprehensive understanding of the different safety types, and their dynamic and seemingly contradictory interactions. As a result, practical implications could be suggested.

A few limitations should be considered as well. First, the study took place within an international and interdisciplinary summer school context in which multiple identities were at stake. An intersectional approach could have provided more detailed and nuanced insights, but it was not part of the scope of this study. Additionally, the absence of male participants in the group may have influenced the results as well. These factors should be taken into account in future studies. Lastly, most of the participants had a basic understanding of the subject, which might have affected their engagement with the topic. We expect that individuals with a more in-depth knowledge of SGSM would engage in discussions with greater clarity and nuance. Nevertheless, since most medical students are likely to have a similar starting point, this study provides valuable insights for SGSM teaching in academic environments.

## Conclusion

Safety emerged as a fundamental cornerstone for effective learning of SGSM, as unanimously expressed by participants. This safety encompassed social, psychological, and cultural dimensions, demonstrating a complex and interconnected relationship. Nevertheless, the restricted perception of safety as comfort and absence of conflict by the participants, led to well-meaning behaviors that paradoxically impeded the attainment of some meaningful learning outcomes. Thus, broadening the understanding of safety and its associated behaviors is imperative for optimizing the learning climate within SGSM educational settings. Challenge and supported divergence are essential elements towards learning and should be enacted as a collaborative effort by students, educators, and institutions alike. Ultimately, fostering a culture of safety within SGSM education holds promise for nurturing inclusive and enriching learning environments conducive to advancing knowledge and practice in this critical domain of healthcare.

## Supplementary Information

Supplementary Material 1

## Data Availability

Availability of data and material: The datasets used and analyzed during the current study are available from the corresponding author upon reasonable request.
