# Supplementary material for: Comfort and challenge in the classroom: exploring the role of safety in sex- and gender-sensitive medicine education
Source: BMC Public Health. 2025 Jul 2;25:2222. doi: 10.1186/s12889-025-23444-2 (PMC12220108; doi:10.1186/s12889-025-23444-2)
Supplement: Supplementary file 1 — Supplementary Material 1 [file 12889_2025_23444_MOESM1_ESM.docx]

**Supplementary file 1: The interview guide**

| **Topics** | **Follow-up questions** |
| --- | --- |
| **Welcome and introduction:**   - Introduction and goal of the interview - Space for questions - Consent to start the audio recording | - Do you consent with this interview? - Do you also consent with audiotaping this interview so we can anonymously transcribe and analyze it? |
| 1. **Safety during the course**  - How is safety important for you as a student of SGSM in this course? | - How do you understand safety? - What does safety feel, look and sound like? |
| 1. **Experiencing safety**  - Can you give an example of a moment when you felt safe this week? - Can you give an example of a moment when you felt unsafe this week? | - What triggered the experience? - What did you notice within yourself (physical, mental, emotional, behavioural) - How did you deal with it? |
| 1. **The importance of safety**  - How is safety important for you as a professional in the field of SGSM? - What are your thoughts about the experience of safety for your patients/respondents in the field of SGSM? | - What makes you feel safe as a professional? Why? - How do you feel you pay attention to feelings of safety for your patients/respondents? Could/should their experience of safety be increased? If so, how? |
| 1. **Barriers and facilitators**  - What helps create safety for you as a student/professional in SGSM? - What hinders feeling safe for you as a student/professional in SGSM? - How do the people in the course help or hinder your feeling of safety in studying SGSM? | - Can you give an example? - What would help you feel safe(r) instead of unsafe? |
| **Addition:**  Is there anything I didn’t ask about when it comes to safety in studying/working in SGSM that you’d like to add to this conversation? |  |
| **Completion:**   - Questions, remarks or additions - Follow-up research - Completion |  |
